# Ubiquitin-Related Modifiers of *Arabidopsis thaliana* Influence Root Development

**DOI:** 10.1371/journal.pone.0086862

**Published:** 2014-01-22

**Authors:** Florian John, Matthias Philipp, Ruth-Maria Leiber, Sanae Errafi, Christoph Ringli

**Affiliations:** Institute of Plant Biology, University of Zürich, Zürich, Switzerland; Umeå Plant Science Centre, Sweden

## Abstract

Ubiquitins are small peptides that allow for posttranslational modification of proteins. Ubiquitin-related modifier (URM) proteins belong to the class of ubiquitin-like proteins. A primary function of URM proteins has been shown to be the sulfur transfer reaction leading to thiolation of tRNAs, a process that is important for accurate and effective protein translation. Recent analyses revealed that the Arabidopsis genome codes for two URM proteins, URM11 and URM12, which both are active in the tRNA thiolation process. Here, we show that *URM11* and *URM12* have overlapping expression patterns and are required for tRNA thiolation. The characterization of *urm11* and *urm12* mutants reveals that the lack of tRNA thiolation induces changes in general root architecture by influencing the rate of lateral root formation. In addition, they synergistically influence root hair cell growth. During the sulfur transfer reaction, URM proteins of different organisms interact with a thiouridylase, a protein-protein interaction that also takes place in Arabidopsis, since URM11 and URM12 interact with the Arabidopsis thiouridylase ROL5. Hence, the sulfur transfer reaction is conserved between distantly related species such as yeast, humans, and plants, and in Arabidopsis has an impact on root development.

## Introduction

Ubiquitins (Ub) are small peptides that allow posttranslational modification of proteins. Ubiquitylation is the reversible attachment of Ub to proteins involving activation, conjugation, and ligation of Ub via corresponding E1, E2, and E3 ligase activities, respectively [1]. While polyubiquitylation targets proteins to degradation via the proteasome, single ubiquitylation has non-proteolytic effects on cellular processes such as transcription, chromatin modifications, or vesicle dynamics. In addition to ubiquitin, a number of ubiquitin-like modifiers are present in most eukaryotes that are also able to tag proteins, usually in a transient manner [2]–[4].

In addition to Ub, ubiquitin-related modifiers (URMs) were identified that are not highly homologous to ubiquitin in respect to the amino acid sequence but share a β-grasp motif as typical structural feature of this type of protein. The primary identified function of URM and URM-related peptides in many different organisms such as archaea, yeast, and eukaryotes is as sulfur carriers in tRNA thiolation [5]–[10]. This process involves activation of URM by an E1-like protein such as Uba4p of yeast which adenylates URMs and transfers sulfur to the terminal glycine resulting in a thiocarboxylate. With the activity of thiouridylases such as Nsc2p and Ncs6p in yeast, the thiol group is then transferred onto uridine residues of tRNAs, a modification which is thought to increase translation efficiency [11]. The function of URMs in thiolation of tRNAs is reminiscent of sulfur transfer reactions in prokaryotes in the synthesis of molybdopterin and thiamine. The MoaD/ThiS proteins involved in this process are not homologous in sequence to URMs, yet also show the β-grasp motif [12]. Hence, URM-type proteins appear to have an activity different from other ubiquitin-related proteins and, because of their similarity to prokaryotic sulfur transfer systems, are considered to be evolutionary intermediates between prokaryotic sulfur transfer and eukaryotic ubiquitin-like protein conjugation systems [13]. In addition to the established role of URM proteins in tRNA thiolation, there is increasing evidence for a second role of URMs in urmylation, a protein modification similar to ubiquitylation in which URMs are conjugated to lysine of target proteins [14], [15].

Recently, two Arabidopsis genes, *URM11* and *URM12*, were identified encoding proteins that are involved in tRNA thiolation [16]. URM11 and URM12 show homology to URM proteins of other organisms and share the β-grasp motif and the terminal di-glycine motif typical for these proteins. In addition to URM11 and URM12, the Arabidopsis homologs of the yeast E1-ligase Uba4p and thiouridylase Ncs6p were identified as CNX5/SIR1 and ROL5, respectively. Both these Arabidopsis proteins have been shown to be involved in the tRNA thiolation process. *cnx5/sir1* mutants show a severe growth defect, whereas the *rol5* mutant is mainly affected in root growth and shows changes in cell wall architecture. The enhanced severity of the *cnx5/sir1* mutant phenotype compared to *rol5* is likely caused by a general defect in sulfur transfer reactions in this mutant that also affects molybdopterin biosynthesis [16]–[19].

This work presents a more detailed characterization of URM11 and URM12 of Arabidopsis. Our data support the finding that URM11 and URM12 are involved in tRNA thiolation. The protein interactions of the URM proteins are conserved in plants, as they interact with the thiouridylase ROL5. Even though *URM11* is expressed to a higher level than *URM12*, there is a significant synergistic interaction between the two proteins. Finally, the analysis of mutants including a *urm11 urm12* double mutant shows that the lack of tRNA thiolation has an effect on the general root architecture but also on root cell development.

## Results

### *Arabidopsis* Possesses Two Proteins with High Sequence and Functional Similarity to Yeast Urm1p

**Figure 1:**
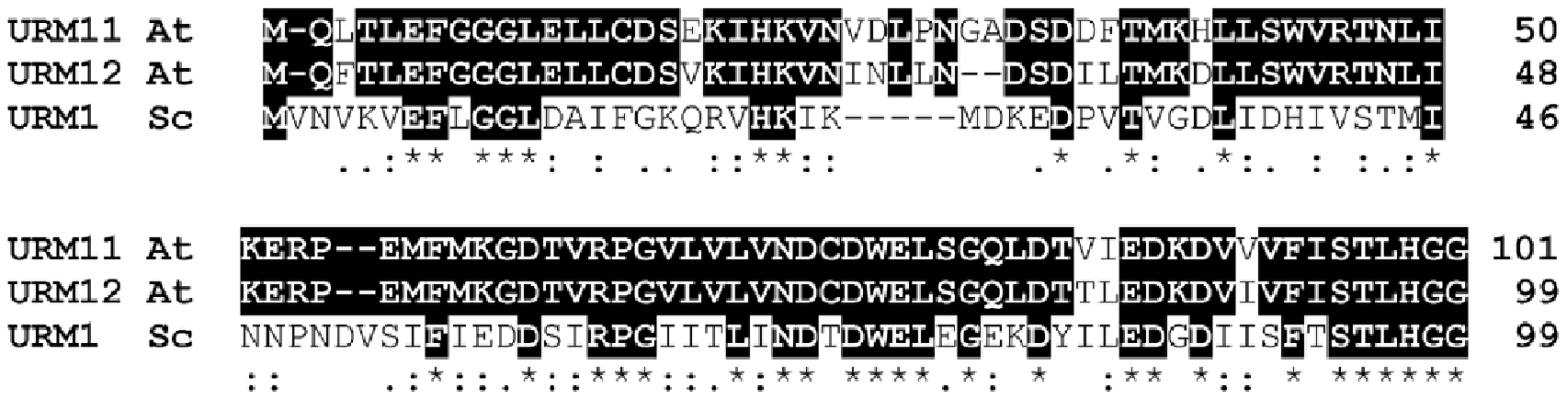
Homology between URM proteins of Arabidopsis and yeast. Alignment of the Arabidopsis URM11, URM12, and the yeast Urm1p. Identical positions are indicated in black, colons indicate conservative amino acid substitutions, and periods indicate similar amino acids.

Proteins with significant homology to ubiquitin-related modifier (URM) proteins have been identified in a number of organisms. The genome of Arabidopsis harbors two *URM* genes, (At2g45695 and At3g61113, respectively) which were termed *URM11* and *URM12* [16]. URM11 and URM12 are homologous to the yeast and human URM proteins, particularly in the C-terminal half including the terminal di-glycine motif essential for URM protein function. This suggests that the C-terminus is less tolerant to variations in amino acid sequence. The two URM proteins of Arabidopsis share high homology to each other with an identity of 87% and a similarity of 91% (Figure 1; [16]).

**Figure 2:**
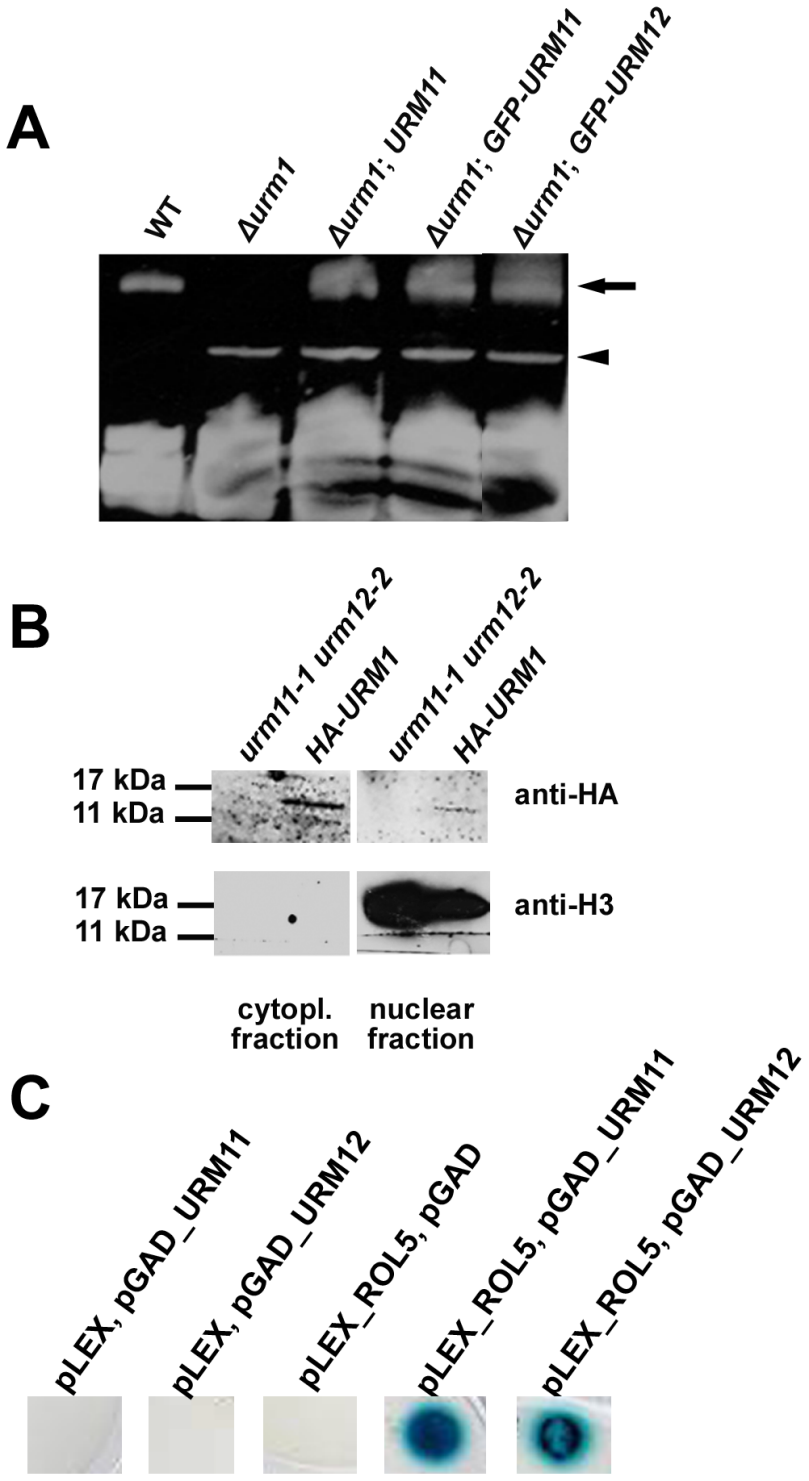
Properties and activities of URM11 and URM12. (A) Bulk tRNA was extracted from wild-type (WT*)*, *Δurm1* and *Δurm1* yeast strains complemented with *URM11* or *URM12*. Thiolated tRNAs (arrow) show slower migration than non-thiolated tRNAs (bottom of gel) in an acrylamide gel containing APM. A band of unknown nature (arrowhead) occasionally occurred. URM11 and the GFP-URM fusion proteins of Arabidopsis are functional in yeast, resulting in tRNA thiolation in the otherwise thiolation-defective *Δurm1* mutant. A representative result of several repetitions is shown. (B) Western blotting of total protein extracts and purified nuclei of *urm11-1 urm12-2* double mutants expressing *HA-URM11* or non-transgenic double mutants. Immunolabelling was done with an anti-HA (upper lane) and an anti-histone H3 (lower lane) antibody. The experiment was performed twice with comparable outcome. (C) A representative result of the yeast-two-hybrid experiment (performed three times independently) revealed the interaction of URM11 and URM12 with ROL5, resulting in yeast growth on selective medium and blue staining of the cells due to β-galactosidase activity. Transformation with only one of two constructs with the empty second plasmid did not result in yeast growth and β-galactosidase activity.

The involvement of URM11 and URM12 in the sulfur carrier process important for the thiolation of eukaryotic cytoplasmic transfer RNAs (tRNAs) was indicated by the successful complementation of the yeast Δ*urm1* mutant defective in tRNA thiolation [8] with *URM11* and *URM12* [16]. To assess whether URM proteins fused to reporter proteins can be used to assess localization of URM proteins, complementation efficiency of the yeast Δ*urm1* mutant by URM and GFP-URM proteins was compared. The Δ*urm1* mutant transformed with cDNAs for *URM11* or *GFP-URM11* and *GFP-URM12* constructs under the control of a constitutively active yeast promoter was analyzed for the presence of thiolated tRNAs. The binding of N-acryloylamino phenyl mercuric chloride (APM) to 2-thiouridine residues leads to the retardation of thiolated tRNAs in acrylamide gels, making them readily detectable. The thiolated tRNAs detectable in wild-type yeast but absent in the Δ*urm1* mutant are present in the Δ*urm1* mutant complemented with either *GFP-URM11* or *GFP-URM12* to an extent that is comparable to the complementation with *URM11* (Figure 2A). This confirms the finding by [16] that the Arabidopsis URM proteins are functional in the sulfur transfer reaction in yeast. Furthermore, this experiment shows that URM11 and URM12 are active with an additional GFP reporter protein at the N-terminus that is considerably bigger in size than the URM proteins.

### URM11 and URM12 Accumulate in the Cytoplasm and the Nucleus

In a next step, we aimed at analyzing the subcellular localization of the Arabidopsis URM proteins. Since URM11 and URM12 appear to be functionally equivalent, URM11 subcellular localization was investigated in onion cells transiently transformed with *35S*:*GFP-URM11* and *35S*:*GFP*. Both the GFP-URM11 fusion protein as well as GFP alone localize to the cytoplasm and show a signal in the nucleus (Figure S1). Since URM proteins are short peptides, it cannot be excluded that the GFP moiety influences protein localization. Therefore, localization was also investigated by an alternative strategy, namely by analyzing the distribution of an HA-URM11 protein in transgenic *urm11 urm12* double mutants (see below). Immunodetection by western blotting of proteins from a total plant extract or a purified nuclear preparation revealed the presence of HA-URM11 in both fractions that migrated at the expected mass of around 13 kDa. In contrast, the histone H3 protein was only detectable in the nuclear preparation and not in the total fraction, confirming a strong enrichment of nuclear proteins in the nuclear fraction (Figure 2B). The detection of HA-URM11 in the nuclear protein preparation suggests that a fraction of URM11 and possibly URM proteins in general localize to the nucleus. This is in line with the data of the high-throughput analysis of protein localization in yeast (http://yeastgfp.yeastgenome.org; [20]).

### Arabidopsis URM Proteins Interact with ROL5

The protein network leading to tRNA thiolation has been investigated in detail in yeast. Within this network, Urm1p interacts with the thiouridylase Ncs6p [7], [8]. To get an insight into the degree of conservation of this network in Arabidopsis, URM11 and URM12 were tested for interaction with ROL5, the Arabidopsis thiouridylase and functional homolog of Ncs6p [19]. To this end, a yeast-two-hybrid experiment was performed using ROL5 as the bait protein and URM11 or URM12 as the prey proteins. For both URM proteins, an interaction with ROL5 was observed as cells grew on selective medium and resulted in GUS activity. Control experiments with the *ROL5*-containing bait vector and an empty prey vector or the empty bait vector with the *URM11*- or *URM12*-containing prey vectors revealed no autoactivation of any of the proteins (Figure 2C). The interaction of URM11 and URM12 with ROL5 provides further evidence for the conservation of the process of sulfur transfer leading to tRNA modification across a wide range of species.

### *URM11* and *URM12* are Ubiquitously Expressed

**Figure 3:**
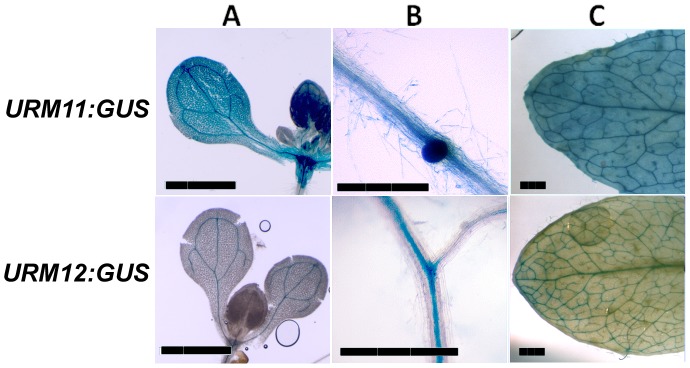
Expression patterns of *URM11* and *URM12*. The expression pattern of both genes was investigated by promoter *GUS* fusion constructs in transgenic Arabidopsis. The *URM11:GUS* construct led to a homogeneous GUS staining at the seedling stage and the adult stage. *URM12:GUS* is predominantly active in vascular tissue. Shoots (A) and roots (B) of seedlings and cauline leaves of adult plants (C) are shown. Bars = 2.5 mm.

According to microarray data of the Genevestigator platform [21], *URM11* and *URM12* are expressed at all developmental stages of Arabidopsis. To investigate *URM11* and *URM12* expression patterns in more detail, the promoter sequences of *URM11* and *URM12* were fused to the *GUS* gene and transformed into Arabidopsis. Several independent transgenic lines were then screened in the T_2_ generation for GUS activity at seedling and adult stage and representative examples are shown in Figure 3. At the seedling stage, the *URM11* promoter induced homogenous *GUS* expression while *URM12* promoter-induced *GUS* expression was mainly detectable in the vasculature. In adult plants, GUS activity was found in most tissues, with *URM11:GUS* resulting in a stronger GUS staining than *URM12:GUS*, which is in agreement with microarray data that found *URM11* to be expressed at a higher level [21]. Again, staining was particularly strong in the vascular tissue. This shows that expression of *URM11* and *URM12* is largely overlapping.

### Mutations in the *URM* Genes Affect tRNA Modification and Root Development

**Figure 4:**
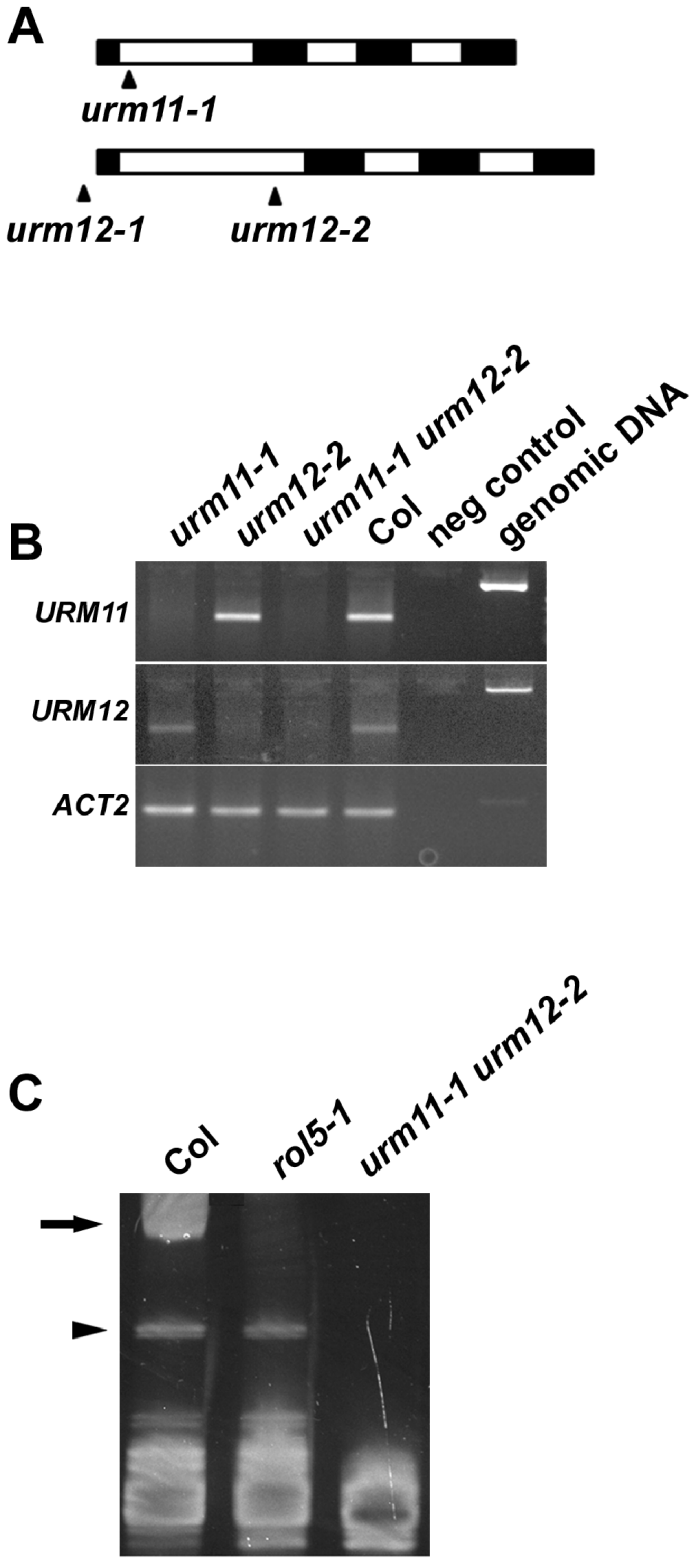
*urm11 urm12* double mutant fails to thiolate tRNAs. (A) Schematic structure of *URM11* and *URM12*. Black boxes represent exons and white boxes introns. T-DNA insertions are highlighted by black arrows and are located in the first intron for *urm11-1* and *urm12-2*, which were further analyzed. (B) RT-PCR on total RNA of entire seedlings revealed absence of *URM11* and *URM12* mRNA in the corresponding mutants. The *ACTIN2* gene was amplified as a control for comparable RNA extraction efficiency. PCR on genomic DNA reveal larger products due to introns. (C) The *urm11-1 urm12-2* double mutant is impaired in tRNA thiolation. In the presence of APM, thiolated tRNAs show slower migration in an acrylamide gel, non-thiolated tRNAs migrate faster (bottom of the gel). In contrast to the wild type, *rol5-1* and *urm11-2 urm12-2* mutants lack thiolated tRNAs (arrow). Bands of unknown nature (arrowhead) occasionally occurred. Representative examples of several independent experiments are shown. Col: wild-type Columbia.

To determine the significance of *URM11* and *URM12* for plant development, T-DNA insertion lines of these loci were identified. For *URM11*, one insertion line (*urm11-1*) was used which contains a T-DNA insertion in the first intron. For *URM12*, two insertion lines were used; *urm12-1* harbors the insertion 250 bp upstream of the start codon and *urm12-2* in the first intron (Figure 4A, allele nomenclature according to [16]). RT-PCR experiments on total RNA extracted from homozygous mutants revealed that the *urm12-1* line still produced *URM12* mRNA (data not shown). By contrast, both *urm11-1* and *urm12-2* lack detectable levels of gene expression of *URM11* and *URM12*, respectively (Figure 4B).

To test whether the absence of *URM* expression has an effect on tRNA thiolation, tRNAs were isolated from wild-type and *urm11-1 urm12-2* double mutant plants. Previously, a strong but not complete reduction in tRNA thiolation has been shown for the *urm11-1* mutant [16]. As a control, tRNA of the *rol5-1* mutant was isolated which was previously shown to lack thiolated tRNAs [19]. A shifted tRNA band, i.e. thiolated tRNAs, were observed only in the wild type but neither in *rol5-1* nor in the *urm11-1 urm12-2* double mutant (Figure 4C), indicating that this tRNA modification is impaired in the absence of URM11 and URM12.

**Figure 5:**
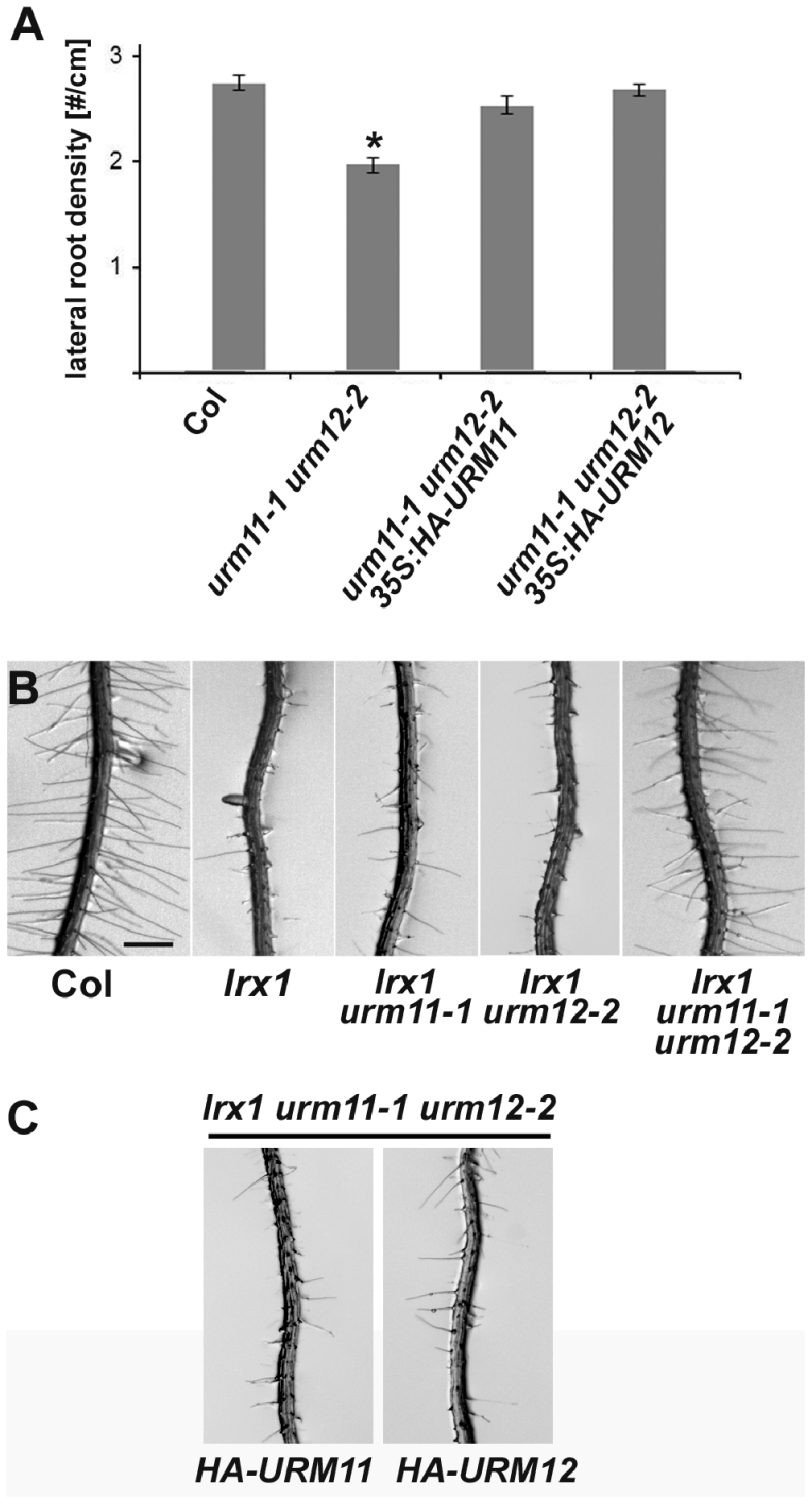
Effects of *urm11-1 urm12-2* on root development. (A) Lateral root density is reduced in the *urm11-1 urm12-2* double mutant compared to the wild type. Complementation with a *35S:HA-URM11* or *35S:HA-URM12* construct restores lateral root formation. Error bars represent the standard error, the asterisk indicates the only value significantly different from the others (two-sided t-test; p = 0.01; n≥25). (B) In contrast to the wild type (Col), *lrx1* mutants frequently have collapsed root hairs. While *urm11-1* or *urm12-2* have no effect on *lrx1*, an *lrx1 urm11-1 urm12-2* triple mutant shows suppression of *lrx1* and develops wild type-like root hairs. (C) Suppression of *lrx1* by *urm11-1 urm12-2* is complemented with either *35S:HA-URM11* or *35S:HA-URM12*, resulting in the *lrx1* root hair phenotype. Col: wild-type Columbia. Bar = 0.5 mm.

To investigate the importance of URM11 and URM12 for plant development, the *urm11-1 urm12-2* double mutant was analyzed, since URM11 and URM12 appear to have a very similar if not identical activity and thus are likely to be functionally redundant. A defect in lateral root development was observed in the double mutant. After growth for ten days, double mutant seedlings had developed a lower density of lateral roots compared to the wild type (Figure 5A). No obvious defect or retardation in shoot development was observed in the double mutant. To test whether the lateral root phenotype is indeed caused by the *urm11-1* and *urm12-2* mutations, the double mutant line was complemented with a *35S:HA-URM11* or *35S:HA-URM12* construct. Complementation with either of the two constructs resulted in wild type-like lateral root formation (Figure 5A), confirming that the absence of URM11 and URM12 induces the reduction in lateral root formation and that both HA-URM proteins are functional.

The locus coding for the URM11 and URM12-interacting protein ROL5 was previously identified as a suppressor of the root hair formation mutant *lrx1* [19]. Since ROL5, URM11, and URM12 are involved in the same process, we explored whether mutations in the *URM* genes have the same effect on *lrx1*. As shown in Figure 5B, root hairs are regularly formed in wild-type seedlings, whereas they are malformed or absent in the *lrx1* mutant. This defect is indeed suppressed in the *lrx1 urm11-1 urm12-2* triple mutant, which showed wild type-like root hair development. By contrast, the *lrx1 urm11-1* and *lrx1 urm12-2* double mutants showed largely an *lrx1* phenotype (Figure 5B). Hence, the *urm11-1* and *urm12-2* mutations synergistically interact, which provides further evidence for URM11 and URM12 having similar functions during root development. The *urm11-1 urm12-2* double mutant, however, did not reveal aberrant root hair morphology compared to the wild type (data not shown). Finally, the *lrx1 urm11-1 urm12-2* triple mutant was complemented with *35S:HA-URM11* and *35S:HA-URM12* constructs. Transgenic lines expressing either of the two *URM* genes developed an *lrx1*-like root hair phenotype (Figure 5C), confirming that the mutations in the two *URM* loci account for suppression of *lrx1*.

## Discussion

Ubiquitin-related modifier proteins (URMs) are found in phylogenetically distantly related species. They can have a low level of identity or similarity, as found for Arabidopsis URM11 and URM12 versus the yeast Urm1p or human Urm1 [16], but are conserved in the β-grasp, a characteristic structure consisting of a core with a pocket of four β-strands and diagonally arranged α-helices, and the C-terminal di-glycine motif [1], [3], [4]. Despite the limited conservation in the primary sequence, Arabidopsis *URM* genes are able to complement the tRNA thiolation defect of the yeast Δ*urm1* mutant ([16]; this work). Our data also show that GFP-URM fusion proteins are functional, which allows a GFP-based analysis of protein accumulation in future studies. In the sulfur transfer reaction, URM proteins interact with a thiouridylase. This interaction is also conserved in Arabidopsis, since URM11 and URM12 interact with the Arabidopsis thiouridylase ROL5, a protein that is essential for the sulfur transfer reaction, as the *rol5* mutant is also defective in tRNA thiolation [19]. In addition to ROL5, URM11 and URM12 have been shown to interact with the E1 ligase SIR1/CNX5 which is important for activation of the URM proteins and essential for tRNA thiolation [16]. Hence, to this point, interactions within the sulfur transfer reaction are well conserved in a diverse range of organisms.

### Mutations in *URM11* and *URM12* Affect Root Development

The thiolation of the uridine in the wobble position of tRNAs conferred by the protein network involving URM proteins is assumed to increase codon-anticodon accuracy, while blocking this tRNA modification is expected to have a negative impact on translation efficiency [11]. However, absence of tRNA thiolation does not have a deleterious impact on the organism. Both the yeast *Δurm1* mutant [5], [22] and the Arabidopsis *urm11-1 urm12-2* double mutant are missing detectable levels of thiolated tRNAs but are viable. However, in both organisms, the growth process is affected. The Arabidopsis *urm11-1 urm12-2* double mutant develops a modified root architecture with a lower lateral root density compared to the wild type. Lateral root formation is under the control of auxin and cytokinin, but is also strongly influenced by nutrient availability [23]–[25]. This is similar to yeast where the mutant *Δurm1* is impaired in pseudohyphal growth, a developmental response to nutrient limitation [22]. Thus, URM proteins of yeast and Arabidopsis, and possibly URM proteins in general, affect processes that are modified by environmental conditions.

In addition to lateral root formation, mutations in *URM11* and *URM12* also affect root hair development. Even though the *urm11-1 urm12-2* double mutant does not show impaired root hair growth, it does suppress the root hair formation mutant *lrx1*. LRX1 is an extracellular protein that is involved in root hair cell wall formation [26]–[28]. The thiouridylase-defective *rol5* mutant was initially isolated as a suppressor of *lrx1* [19], which is the reason why a suppression of *lrx1* was tested in the *lrx1 urm11-1 urm12-2* triple mutant. The comparable effect of *rol5* and the *urm11-1 urm12-2* double mutant suggests that interfering with tRNA modification is causing the suppression of *lrx1*. Since *lrx1* is a cell wall formation mutant, suppression is likely induced by changes in cell wall structures. Indeed, the *rol5* mutant was shown to induce modifications in cell wall structures [19]. A possible mechanism by which changes in tRNA thiolation can affect root architecture and cell wall formation is via the TOR (Target Of Rapamycin) signaling network. The TOR network is a growth controller in eukaryotic cells that senses growth factors and nutrient availability and modulates cellular processes such as translation, ribosome biogenesis, mitochondrial activity, or cytoskeletal dynamics [29]. Alterations in tRNA thiolation modify translation efficiency and have been shown to modulate TOR signaling [11], [22]. In addition, tRNAs are involved in nutritional stress responses via modulating TOR activity [30].

In Arabidopsis, inhibiting the TOR network by rapamycin, a macrocyclic lactone specifically inhibiting the TOR kinase [31], leads to fewer lateral roots, modification of cell wall structures, and suppression of the *lrx1* root hair mutant phenotype [19], [32], [33]. Thus, the phenotypes caused by interfering with TOR signaling by rapamycin treatment are comparable to those observed in the *rol5* and *urm11-1 urm12-2* mutant lines, supporting the hypothesis that alterations in tRNA thiolation have an impact on TOR signaling.

The data presented here support the view that the tRNA thiolation process is conserved across distantly related species. This assumption is corroborated by the analysis of URM protein function in Arabidopsis ([16], and this work) and the interaction of URM11 and URM12 with ROL5, which is equivalent to the interaction of the yeast Urm1p with Ncs6p [8]. A protein-protein interaction requires an overlapping localization of the proteins in the cell. This study revealed that the Arabidopsis URM11 localizes to the cytoplasm and nucleus. Considering the functional similarity of URM11 and URM12, e.g. both being able to complement the yeast Δ*urm1* mutant, it is quite probable that URM12 shows a localization pattern that is comparable to URM11. Since the *HA*-*URM11* construct is under the control of the strong *35S* promoter, a possible ectopic detection of HA-URM11 protein in the nucleus cannot be excluded. Yet, in a high-throughput analysis of yeast protein localization, Urm1p was also localized to the cytoplasm and the nucleus. Even though this localization was later changed to cytoplasmic (manual curation of the localization data), there seems no direct experimental evidence for this restriction (www.yeastgenome.org). The URM11 and URM12 interaction partner ROL5, however, appears to predominantly colocalize with mitochondria [19]. Based on the function of ROL5 in the modification of cytoplasmic tRNAs, ROL5 must, at least transiently, be present in the cytoplasm. Dual localization of proteins in different compartments and organelles is not unusual [34], suggesting that ROL5 is a mobile protein that translocates between mitochondria and the cytoplasm. The biological significance of ROL5 in mitochondria and the URM proteins in the nucleus remains to be elucidated.

Initially, URM-like proteins were expected to serve in a protein conjugation process, comparable to ubiquitylation by ubiquitin [4]. Indeed, target proteins of Urm1p were identified in yeast and human cells where urmylation seems to be induced during oxidative stress [14], [15], [35]. The HA-tagged URM11 and URM12 proteins are functional and will serve as tools in future studies to investigate protein urmylation in Arabidopsis.

## Materials and Methods

### DNA Constructs

For complementation of the yeast *Δurm1* mutant, cDNA clones of the Arabidopsis *URM11* and *URM12* were amplified using the primer pairs *URM11*_for(GGATCCATGCAATTAACTCTTGAATTCGGG)/*URM11*_rev(TTATCCACCATGCAAAGTGGAAAT) and *URM12*_for (GGATCCATGCAATTTACTCTTGAGTTCGGT)/*URM12*_rev (TCATCCACCGTGCAGAGTCGAAAT). The obtained fragments were cloned into pGEM-T easy (Promega) for sequencing. For obtaining the N-terminal GFP fusions, the correct cDNAs in pGEM-T easy constructs were digested with *Bam*HI and a *GFP-Bam*HI cassette [19] was inserted. The resulting clones were digested with *Not*I and cloned into the yeast overexpression vector pFL61 [36].

For the yeast two-hybrid experiment, cDNAs of *ROL5*, *URM11* and *URM12* were amplified with the primer pairs *Kpn*I-*At2g44270-*1F(GGTACCATGGAGGCCAAGAACAAGAAAGCAG)/*Sma*I*-At2g44270*-1R(CCCGGGTTAGAAATCCAGAGATCCACATTG) for *ROL5*, *Xba*I-*URM11*-F (TCTAGAATGCAATTAACTCTTGAATTCG)/*Bam*HI-*URM11*-R(GGATCCTTATCCACCATGCAAAGTGGAA) for *URM11* and *Xba*I-*URM12*-F(TCTAGAATGCAATTTACTCTTGAGTTCGGTGGAG)/*Bam*HI-*URM2*R (GGATCCTCATCCACCGTGCAGAGTCGAAATGAAA) for *URM12*. These fragments were then cloned into pGEM-T easy for sequencing. Subsequently, one of the *ROL5* clones was digested with *Kpn*I and *Sac*I and cloned into pLEXA-N (Dualsystems) cut with the same enzymes. The clones of *URM11* and *URM12* were digested with *Xba*I and *BamH*I and cloned into pGAD-HA (Dualsystems) cut with the same enzymes.

For expressing HA-tagged versions of *URM11* and *URM12*, genomic clones were amplified using the primer pairs *URM11*_*HA*_gen_for (CTCGAGATGTACCCATACGATGTTCCAGATTACGCTATGCAATTAACTCTTGAATTCGGGTAC)/*URM11_HA*_gen_rev(TCTAGAGAAAGGACACTTAAAATTGATAAATACTCTAATATCA) and *URM12*_*HA*_gen_for (CTCGAGATGTACCCATACGATGTTCCAGATTACGCTATGCAATTTACTCTTGAGTTCGGGTACACTATTAC)/*URM12*_*HA*_gen_rev(TCTAGA TCATCCACCGTGCAGAGTCGAAATGAAAACTATA), respectively. For sequencing, the clones were ligated into pGEM-T easy. Correct clones were digested with *Xho*I and *Xba*I and cloned into the expression cassette of pART7 [37], containing a *35S CaMV* promoter and *OCS* terminator, cut with the same enzymes. For plant transformation, the *35S-HAURM-OCS* cassettes were cut out with *Not*I and cloned into the plant transformation vector pBART [38] which is identical to pART27 [37] but contains a gene for resistance to basta instead of kanamycin.

The N-terminal genomic *GFP* fusion constructs for transient expression in plants were produced by *Xho*I digestion of *pART7-HA-URM11* and insertion of an *Xho*I-*GFP* cassette. The *GFP* gene was amplified from the vector *pMDC83* [39] using the primer pair *GFP*_*Xho*I_for(CTCGAGATGAGTAAAGGAGAAGAACTTTTC) and *GFP*_*Xho*I_rev_NOSTOP(CTCGAGGTGGTGGTGGTGGTGGTGTTT).

The *URM11:GUS* construct was produced by PCR amplification of the promoter using the primers *URM11Prom_F* (TTGCGACTCGGATTGGTTAGAATC) and *URM11Prom_R* (CGTCGTTGATGTTTGCAGGAGG) and cloning into *pENTR* (Invitrogen). A correct clone was then used for cloning of the *URM11* promoter into the gateway vector *MDC164* containing the *GUS* gene [39].

For obtaining the *URM12 promoter*:*GUS* construct, 1.8 kb of promoter sequence 5′ upstream of the ATG start codon was PCR-amplified using the primers *URM12Prom_F* (AAGCTTGGTAAATAATCTATAGTTTGTTTAC) and *URM12Prom_R* (TCTAGA CTTCAATGAAGTTTTGCGGTAAC) with an *Hin*dIII site and a *Xba*I at the 5′ and 3′ end, respectively. After digestion with *Hin*dIII and *Xba*I, the fragment was cloned into *pGPTV-Bar* [40] digested with the same enzymes.

### Plant Material and Growth Conditions

All plant lines used are *Arabidopsis thaliana*, accession Columbia. The *lrx1* allele is described in [41]. The *urm11-1* and *urm12-2* allele are the Salk lines 024513 and 070672.47.90, respectively. The *urm12-1* allele not further used in this study is the line ET5108 and is of the accession Landsberg *erecta*.

Seedlings and plants were grown *in vitro* and in soil as described [19]. In brief, seeds were surface sterilized, washed and grown in a vertical orientation with a 16-h-light/8-h-darkcycle at 22°C on plates containing half-strength MS medium. For further growth and propagation, seedlings were transferred to soil and grown with a 16-h-light/8-h-dark cycle at 22°C.

Selection of transgenic plants produced by the standard floral dip method was done on 20 µg/mL basta (for *pBART* and *pGPTV-bar* vectors) or 20 µg/mL hygromycin (*pMDC164*).

### Yeast Strains and Growth Conditions

Yeast strains used in this study were obtained from EUROSCARF, Frankfurt, Germany. The wild-type strain is BY4741 with the relevant genotype MATa; his3Δ 1; leu2Δ 0; met15Δ 0; ura3Δ 0, and the *Δurm1* strain has the relevant genotype BY4741; Mat a; his3D1; leu2D0; met15D0; ura3D0; YIL008w::kanMX4. Yeast strains were grown at 30°C for 2 d on SD plates supplemented with His, Leu, Ade for strains complemented with *pFL61* constructs and His, Leu, Ade and Ura for growth of the wild type.

### Transient Gene Expression in Onion Epidermal Cells

For transient gene expression, onion epidermal cells were transformed by particle bombardment as described [42]. Bombarded tissue was incubated for 1 day at room temperature and the fluorescence pattern was microscopically analyzed.

### Isolation of Nuclear Proteins and Western Blotting

Nuclei were isolated from 1 gram (fresh weight) Arabidopsis 2-week old seedlings grown under sterile conditions following the protocol of [43].

For western blotting, proteins were prepared by mixing nuclear extracts or total seedling material with standard SDS-PAGE loading buffer prior to heat denaturation. SDS-PAGE, blotting onto nitrocellulose, and immunodetection by ECL technology was performed as described by Ringli [44]. Immunodetection was performed using a rat anti-HA antibody (Roche, # 11867423001) and a rabbit anti-histone H3 antibody (Abcam, Ab # 1791), followed by horseradish-coupled goat anti-rabbit and anti-rat antibodies (Santa Cruz Biotechnology, # sc-2004 and sc-2006, respectively), all of which were used in 1∶3000 dilutions.

### Microscopy

Epidermal GFP fluorescence was analyzed using a Zeiss Imager Z1 microscope equipped with an Axiocam HRC. GFP fluorescence of yeast cells was analyzed with a Leica DM6000 equipped with a Leica BFC 350FX. Phenotypic observations and *GUS* expression analysis were done with a Leica LZ M125 stereomicroscope. Data points of lateral root development represent ≥25 seedlings. The experiment was done several times. For the root hair phenotype, over 30 seedlings of each line were analyzed.

### RNA Extraction and RT-PCR

Seedlings were grown vertically on half-strength MS Medium in a vertical orientation for 7 d as described above. The tissue of 120 entire seedlings was frozen in liquid nitrogen and grinded. The powder was then used for RNA extraction using the SV Total RNA isolation System kit (Promega). The reverse transcription was conducted with 500 ng of total RNA using the i_script kit (Biorad). One tenth of the obtained cDNA was then used for RT-PCR using the primer pairs *ACTIN2*F (59-AATGAGCTTCGTATTGCTCC-39) and *ACTIN2*R (59GCACAGTGTGAGACACACC-39), *URM11*_rt_for (ATGCAATTAACTCTTGAATTCG)/*URM11*_rt_rev(TTATCCACCATGCAAAGTGGAA), and *URM12*_rt_for(ATGCAATTTACTCTTGAGTTCG)/*URM12*_rt_rev (TCATCCACCGTGCAGAGTCGAA). The *ACTIN2* PCR was done with 25 cycles,.the *URM* PCRs with 30 cycles of amplification.

### tRNA Extraction and Analysis

Arabidopsis seedlings were grown vertically on plates for 14 d as described. Approximately 250 seedlings were used for extraction. The seedlings were grinded in liquid nitrogen and the material was extracted two times with 8 ml acidic phenol (Sigma), 0.8 ml chloroform and once with 4 ml acidic phenol, 0.4 ml chloroform.

Yeast strains were grown at 30°C in 50 ml liquid SD media supplemented with His, Leu, Ade for strains complemented with *pFL61* constructs and His, Leu, Ade and Ura for growth of the wild type or *Δurm1* mutant. The tRNA was extracted 2 times with 4 ml acidic phenol, 0.4 ml chloroform.

After extraction of the plant or yeast material, tRNA was purified with AX100 columns from MACHEREY NAGEL following manufacturer’s instructions. For analysis, the purified tRNA was separated on an acrylamide gel supplemented with N-acryloylamino phenyl mercuric chloride (APM) by the method adapted from [11].

### Accession Numbers

The Arabidopsis genes discussed in this study have the following accession numbers: *URM11*: At2g45695; *URM12*: At3g61113; *LRX1*: At1g12040; *ROL5*: At2g44270.

## Supporting Information

Figure S1 **Arabidopsis GFP-URM11 localizes to the cytoplasm and the nucleus.** Transient transformation of *35S:GFP-URM11* and *35S:GFP* into onion cells results in a comparable pattern of cytoplasmic and nuclear fluorescence. For each construct, at least 15 transformed cells were analyzed. Bar = 100 µm.
(TIF)
