# Supplementary figures and images for: Ubiquitin-Related Modifiers of Arabidopsis thaliana Influence Root Development
Source: PLoS One. 2014 Jan 22;9(1):e86862. doi: 10.1371/journal.pone.0086862 (PMC3899330; doi:10.1371/journal.pone.0086862)

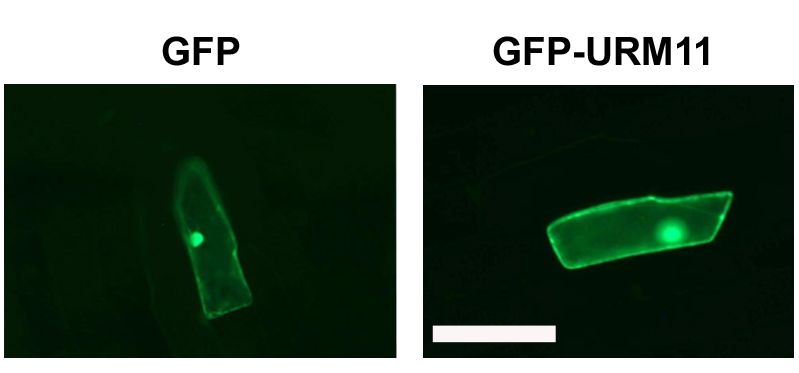

Supplement: Figure S1 — Arabidopsis GFP-URM11 localizes to the cytoplasm and the nucleus. Transient transformation of 35S:GFP-URM11 and 35S:GFP into onion cells results in a comparable pattern of cytoplasmic and nuclear fluorescence. For each construct, at least 15 transformed cells were analyzed. Bar = 100 µm. (TIF) [file pone.0086862.s001.tif]
